# Integrated delivery of family planning and childhood immunisation services in routine outreach clinics: findings from a realist evaluation in Malawi

**DOI:** 10.1186/s12913-020-05571-1

**Published:** 2020-08-24

**Authors:** Jessie K. Hamon, Shari Krishnaratne, Jenna Hoyt, Misozi Kambanje, Shannon Pryor, Jayne Webster

**Affiliations:** 1 Department of Disease Control, Faculty of Infectious and Tropical Diseases, London School of Hygiene & Tropical Medicine, Keppel Street, London, WC1E 7HT UK; 2 Save the Children International, Blantyre, Malawi; 3 Save the Children US, Washington, DC USA

**Keywords:** Malawi, Realist evaluation, Integration, Family planning, Childhood immunisation, Outreach clinic, Accessibility

## Abstract

**Background:**

Family planning (FP) needs among postpartum women in low- and middle-income countries remain largely unmet. Integrating FP with childhood immunisation services could partially reduce this unmet need by creating multiple opportunities for timely contact with FP services during the 12 months following childbirth.

**Methods:**

A realist evaluation of an intervention integrating FP and childhood immunisation services in routine outreach clinics in two rural districts of Malawi was conducted. A Context-Mechanism-Outcome (CMO) framework was used to describe the drivers of the intervention. A detailed programme theory was developed based on the analysis of semi-structured interviews and focus group discussions with 50 stakeholders.

**Results:**

A total of 9 core mechanisms were identified, which centred on constructs of access. Findings revealed that on the demand side, women were motivated to attend outreach clinics due to shorter travel distances; they felt confident they could access FP services and use contraceptive methods covertly if needed; and when supported by their husband, they were empowered to take up the use of contraceptive methods. On the supply side, providers were empowered through the training they received to provide integrated services; they were confident in their ability to provide essential services; and they were motivated by teamwork and by the recognition they received for their work. Additionally, some providers were found to be unwilling to walk long distances to reach remote clinics, which was seen to negatively affect the availability of services.

**Conclusions:**

The delivery of integrated FP and childhood immunisation services in the context of routine outreach clinics in rural Malawi was seen to trigger mechanisms of accessibility and to improve the acceptability and availability of FP services. However, further research is needed to understand how the integration of these services in a routine outreach clinic setting may affect other dimensions of accessibility, including the approachability, appropriateness and affordability of services.

## Background

As highlighted in target 3.7 of the sustainable development goals (SDGs),^1^ universal access to high-quality family planning (FP) services is essential to improve the health of women and their children [1]. Access to quality FP services is particularly important for women during the twelve months following childbirth, or extended postpartum period, as adequate birth spacing has been shown to reduce the risks of miscarriages, preterm births, stillbirths, and child and maternal morbidity and mortality [2–4]. Nevertheless, FP needs among postpartum women in many sub-Saharan countries remain largely unmet, resulting in a high prevalence of unintended pregnancies during the twelve months following childbirth [5–8].

In Malawi, the distance to health facilities (on average 10–15 km instead of the Ministry of Health recommended 5–8 km), the absence of FP commodities due to supply-chain issues, the shortage of trained FP providers, and the lack of support from community leaders are some of the factors that contribute to the unmet need for FP [9]. According to the 2015–2016 Demographic and Health Survey (DHS), the unmet need for modern contraceptive methods (MCMs) among married women in Malawi is 18.7%, with the unmet need among women in rural areas higher than in urban areas [10]. Nationally, Malawi is in the final stage of contraceptive use growth on the S-Curve, and therefore equity in contraceptive use among population sub-groups is a priority [11]. In light of this, the Government of Malawi identified strategic activities in the country’s 2016–2020 Costed Implementation Plan for FP to improve FP services [9]. Among these activities is the integration of FP with other health services, including childhood immunisation programmes. By creating repeated opportunities to expose women to FP services during the year following childbirth, the integration of FP and childhood immunisation services is seen as a way of improving contraceptive use among postpartum women whilst also optimising the use of limited resources.

Given Malawi’s high national childhood immunisation coverage (above 90% for most antigens), the integration of these services could serve to effectively broaden the reach of FP services [12]. Though recognised as a promising high impact practice for FP service delivery, little is known about the integration of FP and childhood immunisation services in practice [13]. Evidence from a randomised controlled trial in Rwanda suggests that this type of integration can significantly increase contraceptive use among postpartum women without hindering childhood immunisation uptake [14]; however, a more comprehensive understanding of this complex intervention is needed. This evaluation sought to contribute to this knowledge gap by exploring the components and drivers of a non-governmental-organisation-led intervention in which these two services were integrated. Overall, the evaluation aimed to synthesise the experiences of those involved in, or targeted by, the intervention and to develop a programme theory based on the contextual triggers and mechanisms of the intervention.

## Methods

This study was part of a multi-country realist evaluation. The overall evaluation included several stages, which are described in Krishnaratne et al. [15]. In Malawi, a realist approach was used to identify and examine the mechanisms and their contextual triggers that drive the outcomes of integrated FP and childhood immunisation services in routine outreach clinics where the intervention was implemented. Here, mechanisms refer to the decisions that people make in response to the intervention, which are influenced or triggered by a particular context.

First developed by Pawson and Tilley in the 1990s, realist evaluation is a theory-driven and process-focused method [16]. This approach to evaluation is centred on the belief that to be useful for policy makers, evaluations should identify “what works for who, in what respects, to what extent, in what contexts, and how” [16]. It uses a context-mechanism-outcome (CMO) framework to understand specific elements of context triggered mechanisms that drive outcomes of the intervention, and it can be used to test and refine programme theories as well as to test an intervention in a particular setting.

### Study setting

The intervention was initiated in January 2015 in 24 routine outreach clinics across three districts where the implementing organisation already supported immunisation programmes: Thyolo, Mwanza and Blantyre (rural areas only). Prior to the intervention, childhood immunisations were provided in routine outreach clinics, as part of the Government of Malawi’s Expanded Program on Immunisation, to reach communities located more than 5 km from a health facility. The intervention aimed to leverage these outreach clinics and the high childhood immunisation coverage in these districts, where 80.0 to 91.3% of children aged 12–23 months receive all basic vaccinations [10], to increase MCM use by integrating the delivery of FP services with childhood immunisations.

In these districts, as is the case in Malawi’s rural areas more generally, most women access FP services through public health facilities [10]. These services are provided by Nurses in health centres where injectables, implants, condoms and oral contraceptive pills are available, and by community-based health workers, such as Health Surveillance Assistants (HSAs). HSAs are trained to counsel women on all MCMs, but only provide short-term methods such as injectables, condoms, and oral contraceptive pills [9]. They also refer women preferring other MCMs to the nearest health facility. According to the 2015–2016 DHS, the rate of MCM use in these districts ranges from 58.7 to 60.3%. The most commonly used MCM is injectables, with its use among married women ranging from 30.2 to 35.8% across the three districts, and the unmet need for FP is quite consistent ranging between 18.7 to 18.9% [10] among married women. Slightly more of the unmet need relates to the desire to limit births (10.2 to 10.6%) rather than to the desire to space births (8.1 to 8.6%). Though district-level figures for sexually active unmarried women aren’t captured by the DHS, nationally, 39.8% of these women have an unmet need for FP [10].

### The intervention

The intervention had four objectives: 1) improve the capacity of providers to deliver FP services; 2) improve the retention of clients; 3) improve the availability of FP and immunisation supplies; and 4) raise awareness in communities about the benefits of MCM use.

**Fig. 1:**
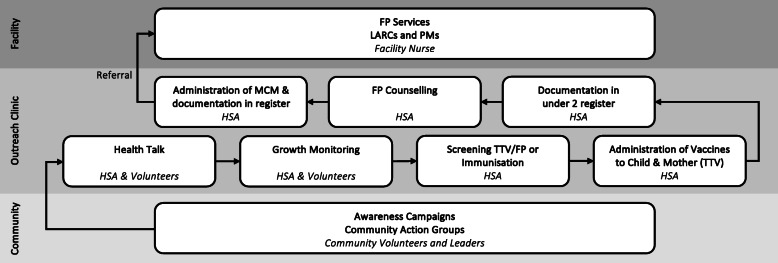
Intervention model for integrated FP and childhood immunisation services

Though the intervention encompassed a multi-level model (Fig. 1), the central component was the combined provision of FP and childhood immunisation services at monthly routine outreach clinics using a defined and deliberate client flow model. Outreach clinic services were provided in an existing building (school or church) or were delivered in an open space (under a tree) if a building was not available. Each outreach clinic was staffed with 3 to 8 HSAs who were trained to provide all services offered and who were encouraged to rotate roles at each site to be comfortable performing every role involved in the client flow model. Several community members also volunteered in each outreach clinic to support HSAs.

According to the intervention’s design, the client flow and information about child development, nutrition, exclusive breastfeeding, FP and immunisations were explained to women during a group health talk at the start of the clinic day. Children then underwent growth monitoring (measuring and weighing). Women and children were screened for vaccinations and FP needs. Eligible women received the Tetanus Toxoid Vaccine (TTV) and children were immunised according to their individual schedule. Women who were new or repeat FP users participated in a compulsory FP counselling session before receiving the MCM of their choice. MCMs offered in outreach clinics included oral contraceptive pills, injectables and condoms. Women choosing to take up a permanent method (PM) or long-acting reversible contraceptive (LARC), such as intrauterine devices or implants, were given a written referral to the closest health facility offering these methods.

According to the intervention’s monitoring data, numerous women benefited from the integration of FP and childhood immunisations. In the months leading up to and including this evaluation (January 2016 to March 2018) 17,381 MCMs were supplied to women in the intervention’s outreach clinics. Among these MCMs, most were injectables (96.42%), followed by oral contraceptive pills (2.74%) and condoms (0.84%).

### Formulating an initial programme theory

A three-step process was used to define the initial programme theory. First, an operational theory was developed by the evaluation team based on a review of intervention documents to outline how intervention components were intended to work. Second, the operational theory was shared and discussed with intervention designers and implementers during a 2-day workshop. The feedback they provided about the operational theory along with the assumptions they held about the response of actors in the intevention were combined to draft the initial programme theory. Third, the initial programme theory was refined by the evaluation team and was then used to inform the collection of empirical data.

### Empirical data collection

Semi-structured interviews (SSIs) and focus group discussions (FGDs) were conducted with key stakeholders in two (Blantyre and Thyolo) of the three intervention districts. The intervention’s third district was excluded from the data collection due to logistical constraints. Specifically, the study team was unable to reach the intervention sites in Mwanza given the resources and time available for data collection.

Purposive sampling was used to select key stakeholders who were either involved or had an interest in the intervention, which included the intervention team, government administrators, health service providers, community leaders and women. Participants were selected with input from the intervention team using a maximum variation sampling approach to include a range of knowledge and perspectives about the intervention [17]. Providers that were selected had experience in delivering services in outreach clinics where the intervention was perceived to be well or less-well received according to project monitoring data.

Interview and discussion guides were developed for SSIs and FGDs with themes informed by the initial programme theory and focused on: the socio-cultural and health systems contexts; the delivery of the intervention in general and the services more specifically; the women’ and providers’ decisions; and the women’s past and current use of MCMs. Questions relating to the initial programme theory were also included in the guides. A member of the evaluation team and a local research assistant led the interviews. All interviews were conducted in either Chichewa or English, and were audio recorded, transcribed *verbatim* and translated into English by an experienced translator.

### Data management and analysis

The translated transcripts from the SSIs and FGDs were imported into NVivo 11.2 for coding and analysis. Quotes were anonymized; however, the type of stakeholder corresponding to each quote was retained to aid analyses. The data was coded by a single investigator and codes were discussed with the evaluation team throughout the data analysis to ensure a consensus was reached where ideas and opinions differed.

Deductive coding was conducted using a framework based on the initial programme theory and the interview guides’ themes. The coding framework included: 1) the actors involved in the delivery and use of FP; 2) the socio-cultural and health systems contexts; 3) the delivery of the intervention; 4) the decision-making of health service providers and women; and 5) the intervention outcomes relating to FP uptake. An inductive approach was subsequently used to code emerging themes and sub-themes derived from the data. Coded quotes were then scrutinised and CMO configurations were identified. An iterative process was used to construct CMOs and to refine the programme theory, as these were developed in tandem. Careful consideration was given to the decision-making of women and providers and to the contexts that triggered these decisions. Once agreement was reached by the evaluation team on what constituted the core CMO configurations and the revised programme theory, example quotes were extracted to illustrate and enrich both.

Finally, a review of published theories on access to health services was conducted due to the importance of this concept within the initial programme theory. Through this review, the Access to Health Care framework was selected to help explain the core CMOs [18]. This person-centred framework provided a holistic conceptualisation of access by presenting it as a function of supply and demand determinants, and by defining it in terms of five ‘accessibility dimensions’ (approachability, acceptability, availability and accommodation, affordability, and appropriateness) and their ‘corresponding abilities of persons’ (ability to perceive, to seek, to reach, to pay and to engage) [18].

## Results

### Initial programme theory

**Fig. 2:**
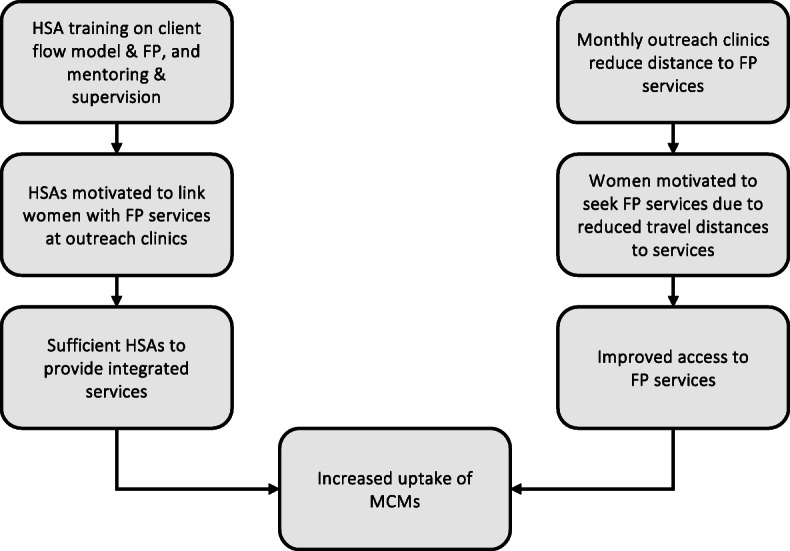
Initial programme theory

The initial programme theory (Fig. 2) suggested that the training of HSAs in the integrated delivery of FP services, together with their mentoring and supervision, was key in motivating HSAs to link women with FP services at outreach clinics. Once women were linked to FP services, the presence of sufficient HSAs to provide FP services was expected to be a major driver of intervention outcomes. It was also thought that the use of outreach clinics would reduce the distance travelled by women to access services, in turn motivating them to seek FP services.

### Empirical data

A total of 22 SSIs and 5 FGDs were conducted between February and March 2018 and later analysed. These were conducted in communities and at routine outreach clinics in Thyolo and Blantyre districts (*N* = 50). Stakeholders included intervention team members (*n* = 2), government health administrators from the district and national levels (*n* = 12), health service providers (*n* = 27), community leaders (*n* = 2) and women who self-reported as either using or not using MCMs (*n* = 7).

### Contexts

According to respondents, the context defining the demand for FP services was characterised by issues of access to health services and by dwindling household resources in communities. First, the distance to health facilities was found to impede women who wanted to access FP services. Respondents explained that women prioritised seeking essential services over what they perceived as non-essential services. Though women thought of childhood immunisations and growth monitoring as indispensable, in most cases FP was not considered similarly essential. Second, the context of dwindling household resources due to rising deforestation in the area coupled with food scarcity negatively influenced, for some, the value traditionally attributed to large families. For example, respondents mentioned witnessing a shift in the demand for MCMs among families whose livelihoods were affected by rising levels of deforestation.

Similarly, the context in which services were provided was also characterised by issues of access. According to respondents, Malawi’s health policies state that each HSA should be tasked with providing services to at most 1000 people and should ideally live in the catchment area they serve. However, respondents reported examples of HSAs catering to two or three times the expected number of people. Also, due to the remote nature of certain communities and a lack of accommodation, many HSAs reportedly lived outside the area they served, meaning they travelled long distances to reach the catchment area they served and even further to reach isolated communities within the area. Respondents emphasised that the remote nature of the rural communities contributed to the relatively high number of HSAs chosing to work in urban facilities, resulting in certain communities being further underserved.

### Mechanisms

Nine core mechanisms were identified through this evaluation, all of which centred on access. Four mechanisms corresponded to demand-side-factors of access to healthcare and five corresponded to supply-side-factors. Here, demand-side-factors are defined as those determining the need and use of health services among community members, whilst supply-side-factors are those influencing the capacity of providers and outreach clinics to deliver such services. These mechanisms, their contextual triggers and the outcomes they drive are presented below along with demonstrative quotes from interviews and FGDs.

#### Demand-side mechanisms

##### Mechanism 1: women are motivated to attend outreach clinics due to shorter travel distances.

The context created by the intervention of monthly community-based provision of integrated FP services in routine outreach clinics was perceived by respondents to motivate women to seek FP services due to the relatively short travel distances to the clinics. This was believed to increase access to FP services, resulting in improved MCM uptake.

*“I get the* [Depo-Provera] *injection together with my friends. They say they can’t stop, seeing as the access point is near. So we will continue accessing.”* Woman MCM user_7

##### Mechanism 2: women feel confident that they can access FP without their husband knowing.

By integrating FP and childhood immunisation services, a context was created in which women could access FP services without their husband’s knowledge. For women with a husband that was not supportive of FP, this component of the intervention provided them with the confidence to covertly access FP services, which was perceived as ultimately leading to improved access to FP services.

*“Not all husbands would accept that their wives should take family planning methods, but when the women say ‘I’m just going to the under 5 clinic and she finds and uses the family planning methods there, the husband may not notice that she has also accessed this service … the husband will think it is about the vaccines.”* Health administrator_19

*“Some women are not allowed to access family planning by their husbands … With the integration, they can come for immunisation while their own health passport is in the pocket, accessing family planning along with the child’s immunisation.”* HSA FGD_3

##### Mechanism 3: women choose discreet MCMs.

Husbands’ support of FP emerged as an important contextual trigger for the choice of MCM among women accessing services in routine outreach clinics. Interviews with providers revealed that women with husbands who were not supportive of FP typically chose to adopt a discreet or non-visible MCM, increasing the demand for injectables (Depo-Provera).

*“Most of them use the injectable one because of the husbands. For example, if they use implants that means husbands will discover – then there’s a problem. If they take pills, then the same husbands will discover – problems.”* Nurse_22

*“But for those, for many women who are taking Depo-Provera, they say it’s more secretive, even from their husbands or from other women*.” Community Nurse_9

##### Mechanism 4: women feel confident to accept MCMs when these are offered.

Husbands who reportedly supported FP emerged as a separate contextual trigger. In particular, women and community members linked the financial benefits associated with child spacing and having fewer children to husbands’ support for FP. This context elicited confidence among women who felt empowered to accept MCMs when these were offered to them. It was reported that for many women this confidence translated into same-day MCM uptake in outreach clinics.

*“There is scarcity of resources, like land and even paying school fees. If you have so many children, you cannot afford to pay their school fees.”* Village chief_10

*“*[My husband] *sees that when I am pregnant or when I have a baby, the budget is stretched as opposed to when I don’t have a baby. Because even when one is pregnant, budgets change, and more when there is a baby. So, when we don’t have any baby what we do is plan for the children, what we need to do for them. Therefore, our home is organised.”* Woman MCM user_14

#### Supply-side mechanisms

##### Mechanism 5: HSAs are empowered by the knowledge they gain about how to administer injectables.

The training received by HSAs in this intervention on FP counselling and the administration of short-term MCMs, particularly injectables, was perceived to be a key intervention component. This training was seen as empowering HSAs by giving them the knowledge needed to administer MCMs, which resulted in the availability of injectables at outreach clinics.

*“HSA1: After the training … that’s when we started to do the real work. HSA2: [Before the training] we were just referring the clients when they said that they want Depo* [injectables]*.”* HSAs 1 and 2 FGD_1

*“Very empowered. Whenever I visit them* [HSAs] *they don’t have a lot of complaints. But they now start giving me a lot of suggestions on how the programme can run better … They are very well empowered.”* Health administrator_18

##### Mechanism 6: HSAs are confident they can provide services that are needed due to the client flow model.

The HSAs’ use of the intervention’s client flow model to organise their services and team emerged as another key intervention component. Due to the client flow model, HSAs felt confident that they could provide the FP, childhood immunisation, and growth monitoring services needed by women and children living in underserved communities. The HSAs’ confidence enabled the integrated provision of services in routine outreach clinics.

*“In the past we had problems with the client flow, we had no client flow and there was no order at the clinic. We would miss out some of the children because they were not recorded … But after we were trained, we were able to capture each and every child because they now move in order. We know that from weighing, they go for screening and from screening they go for immunisation, then from there they go for registration and off they go. So to us it was very good.”* HSA FGD_1

##### Mechanism 7: HSAs and volunteers are motivated by teamwork.

In the context of limited health service providers working in rural areas, HSAs felt supported by volunteers in outreach clinics who helped them maintain the clinics’ client flow. This was seen as prompting teamwork among HSAs and volunteers, which motivated them to deliver integrated services.

*“It has also helped in bringing coordination and team work among HSAs. At first we weren’t doing things in order, anyone who wanted to go for outreach would go, who doesn’t want, wouldn’t go. As of now, we work as a team and in great coordination … So since the beginning of this project,* [we] *are still doing work as a team … among the HSAs and the volunteers.”* HSA FGD_4

##### Mechanism 8: HSAs are motivated by the feeling that their work is valued.

Respondents reported that many women attended routine outreach clinics. For instance, a health administrator emphasised that there were at times upward of 300 women waiting to receive services in one day. This high demand for services, combined with the ability to maintain the clinic’s client flow through the support from volunteers, resulted in HSAs feeling their work was valued by communities, which in turn motivated them to deliver integrated services in routine outreach clinics.

*“*[The clients] *are many, so it’s really encouraging, and it gives us feedback that they really received the services very well. So, for us, as providers, we are really encouraged that the services are being given to the right people.”* Nurse_9

*“*[HSAs] *have been encouraged by the turn up of the clients* [at outreach clinics]. *Because if they go there without getting the clients, they could be reluctant to go again because there could be no one to get the services…”* Health administrator_16

##### Mechanism 9: some HSAs are unwilling to walk long distances to reach remote outreach clinics.

The remote location of outreach clinics, combined with transport issues, meant that HSAs often had to walk long distances to reach the clinics. This was seen as a barrier for some HSAs and no intervention component was implemented to address this at the time of this evaluation. Respondents stated that some HSAs were unwilling to walk long distances, which was seen as negatively affecting the availability of providers and resulting in understaffing of the most remote or difficult to reach clinics.

*“Transportation and tracking of the HSAs is another challenge … They can’t carry all those things* [supplies]. *They need reliable transport to move from one place to another.”* Health administrator_17

*“Some HSAs are hard-working. You can see they sacrifice themselves to walk, but others say ‘I don’t have transport. I don’t want to walk’. So, some are committed, and others are not so there is a lot of variation in commitment. So, if people are committed like that, we need to find a way to incentivise them to do more.”* Health administrator_27

### Revised programme theory

The initial programme theory was revised in light of the nine core mechanisms that emerged from the empirical data analysis. The theory was also extended beyond the CMO configuration to include two additional categories that were considered separate yet crucial to the explanation of reported outcomes: 1) the intervention components (I) that stood out from the overall context and prompted the core mechanisms; and 2) the actors (A) who enacted the mechanisms. These were highlighted separately, similarly to the approach used by Dossou et al., [19]. The revised programme theory (Table 1) therefore encompassed context-intervention-actor-mechanism-outcome (CIAMO) configurations.

**Table 1:** Revised programme theory with CIAMO configurations

Context	Intervention Component	Actor and Mechanism	Outcome
**Demand-side**			
Underserved communities in hard-to-reach areas (C)	Monthly provision of integrated FP services in routine outreach clinics (I)	Women (A) are motivated to attend outreach clinics due to relatively short travel distances (M1)	Increased access to FP services (O)
Some husbands are not supportive of FP (C)	Integration of FP and childhood immunisation services (I)	Women (A) feel confident that they can access FP services without their husband knowing (M2)	
		Women (A) choose discreet MCMs (M3)	Women opt to use injectables (O)
Some husbands support birth spacing for financial reasons (C)	Integration of FP and childhood immunisation services (I)	Women (A) feel confident to accept MCMs when these are offered (M4)	Same-day uptake of MCMs (O)
**Supply-side**			
The health services needed by women and children are not available in underserved communities (C)	HSAs are trained on the outreach clinics’ client flow, and on childhood immunisation and FP services (I)	HSAs (A) are empowered by their knowledge of FP injectables (M5)	HSAs provide FP injectables (O)
		HSAs (A) are confident they can provide the services needed (M6)	Integrated services are provided at outreach clinics (O)
Limited health service providers work in rural areas and a high demand for services in routine outreach clinics (C)	Volunteers support HSAs in maintaining the client flow in outreach clinics (I)	HSAs and volunteers (A) are motivated by team work (M7)	
		HSAs (A) are motivated by feeling their work is valued and recognised (M8)	
Routine outreach clinics are located in hard to reach areas (C)	No defined intervention (I)	Some HSAs (A) are unwilling to walk long distances to reach remote outreach clinics (M9)	Understaffing in some outreach clinics (O)

### Synthesis

The nine core mechanisms identified in this evaluation were found to drive the accessibility of FP services. In particular, these mechanisms centred on two accessibility dimensions (acceptability, and availability and accommodation of services) and on their corresponding abilities (seeking and reaching healthcare services) as defined in Levesque et al.’s framework [18]. Other dimensions included within this framework – namely, the approachability, appropriateness and affordability of healthcare – were not found among the core mechanisms triggered by the intervention.

**Fig. 3:**
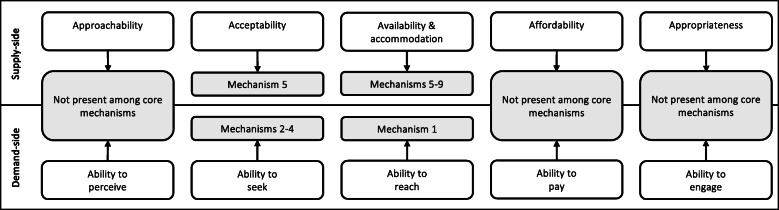
Access to Health Care framework [18] with mechanisms of accessibility

As presented in Fig. 3, mechanisms of acceptability included: (M2) women feeling confident that they can access FP without their husband knowing; (M3) women choosing non-visible MCMs; (M4) women feeling confident to accept MCMs when these are offered; and (M5) HSAs being empowered by their knowledge of FP injectables. Mechanisms of availability and accommodation included: (M1) women being motivated to attend outreach clinics due to the relatively short travel distance and time; (M5) HSAs being empowered by the knowledge they gained about how to administer injectables; (M6) HSAs feeling confident that they can provide the services women and children need; (M7) HSAs and volunteers being motivated by teamwork; (M8) HSAs being motivated by the feeling that their work is valued; and (M9) some HSAs being unwilling to walk long distances to reach remote outreach clinics.

## Discussion

Despite being recognised as a promising approach to reducing the unmet need for FP among postpartum women, few studies have investigated the integrated delivery of FP and childhood immunisations. This study aimed to generate insights about the integrated delivery of these two services by examining the drivers of an outreach-based intervention in two districts of Malawi. A realist approach was used in this evaluation as it allowed for the complexity of the intervention to be captured. The findings have programmatic implications for the intervention studied and could be used to make inferences on the transferability of the intervention as it relates to specific contexts.

The initial programme theory was revised to incorporate the mechanisms that emerged through this evaluation. Though more detailed, the revised programme theory held many of the initial theory’s key elements. This is likely because the intervention team, who contributed to the development of the initial theory, had a practical understanding of the intervention’s drivers based on their experience implementing the intervention and supporting healthcare delivery more generally in the targeted districts. For instance, the initial theory suggested that training, mentoring and supervision were crucial in motivating HSAs to deliver integrated services. Findings revealed that HSAs’ confidence and motivation was primarily derived from their training, but also from the recognition they received for their work, rather than from their mentoring or supervision. This is consistent with well-documented evidence that links community-based health worker motivation to their training and the recognition they receive from the communities they serve [20–22].

Furthermore, the initial theory’s emphasis on the sufficient availability of HSAs to provide FP services and on the reduced distances travelled by women due to the proximity of outreach clinics were also reflected in the findings. However, whilst the intervention was found to improve women’s ability to reach FP services by reducing travel distances, it had the inverse effect on HSAs who were hindered by the remoteness of some outreach clinics and by transport issues. Similar findings were reported in a 2014 case study of integrated delivery of maternal and child health services in Malawi’s central region health centres [23]. The case study revealed that whilst clients were satisfied with integrated services compared to stand-alone services, the providers delivering these services in rural health centres viewed their expansive catchment areas and the lack of suitable housing near facilities as barriers to their work. Designing an intervention component to overcome access challenges encountered by HSAs could help address understaffing in these clinics and further improve the availability of FP services.

Finally, an unanticipated effect of the intervention that was absent from the initial programme theory was how it helped women partially overcome the barriers associated with a lack of support for FP from their husband. In particular, women’s ability to engage in covert MCM use due to the integrated nature of the FP services and the availability of discreet MCMs at oureach clinics was seen to increase their decision-making autonomy and to improve their use of MCMs. These findings are further discussed in Hoyt et al.'s “As a woman who watches how my family is…I take the difficult decisions”: integrated family planning and childhood immunisation services in five African countries (submitted) and are consistent with results from other studies, which highlight the link between improved female decision-making autonomy and MCM use [24–26]. However, the benefits of enabling covert MCM use should be weighed against potential risks such as delayed treatment seeking behaviours and non-adherence to medical interventions when complications from MCMs arise, as well as domestic violence when covert MCM use is uncovered [27, 28].

Overall, results suggest that the integration of FP and childhood immunisations in routine outreach clinics can improve the acceptability and availability of FP services within the specific contexts in which the intervention was implemented. However, further research is needed to explore how other dimensions of accessibility are driven or inhibited by the intervention’s model to yield a more detailed understanding. For instance, investigations that capture the appropriateness of integrated FP services in terms of the fit between services and clients’ needs, and the quality of care are needed [29]. Such studies, combined with the ground work layed by this evaluation could be used to inform initiatives, such as those proposed by the Government of Malawi, to effectively integrate the delivery of FP and childhood immunisation services.

## Limitations

In the interpretation of the evaluation’s results, the purposive selection of respondents represents a potential limitation in understanding the breadth of the intervention and to the generalisability of the study findings. Respondents were selected to include a wide range of experiences and perspectives on childhood immunisations, FP, the community and the health systems based on the initial programme theory. However, it is possible that key perspectives may have been overlooked given the limitations of this sampling approach. For instance, some concepts that were considered relevant to the programme theory emerged inductively through the analysis of empirical data, which suggests that a broader approach to selecting respondents may have helped better understand the intervention. This limitation is, however, less concerning given that respondents were identified according to their ‘CMO investigation potential’ [16] and priority was given to the views of the intervention’s clients (women) and implementers (government administrators and health service providers), as they were believed to be best positioned to provide information about the programme theory. Additionally, though the views of husbands were described by respondents, the absence of their own voices could be considered a limitation. Moreover, the inclusion of the third intervention district (Mwanza) in the study may have provided a more comprehensive understanding of the intervention; thus, it’s exclusion from the data collection could be considered a further limitation. Finally, the use of a single investigator to code the qualitative data may have introduced a personal bias and prevented the recognition of key themes. However, the use of a coding framework based on the initial programme theory and the inductive addition of themes during the coding process resulting from the evaluation team’s discussions about the codes, are likely to have helped mitigate this potential bias.

## Conclusions

Findings from this evaluation suggest that the integration of FP and childhood immunisation services in a routine outreach clinic setting can improve the acceptability and availability of FP services. In a context where women’s geographical access to FP services is constrained and where communities, particularly husbands, are more or less supportive of FP use, interventions that are designed to boost women’s confidence, provide discreet MCMs, and reduce the distance between households and services can improve access to FP services and uptake of MCMs. Additionally, in a context of high demand for services and hard-to-reach health facilities, using a client flow model devised to streamline the delivery of integrated services, delivering services in a routine-outreach-clinic setting, and training HSAs to deliver integrated services alongside community volunteers can motivate HSAs to confidently deliver integrated services. Further research is needed to understand how the integration of these services in a routine-outreach-clinic setting may affect other dimensions of accessibility, such as the approachability, appropriateness and affordability of services. Additional studies are also needed to measure the impact of the delivery of integrated FP and childhood immunisation services on MCM uptake.

## Data Availability

The datasets used and/or analysed during the current study, as well as the data collection instruments, are available from the corresponding author on reasonable request.

## Abbreviations

A: Actor
C: Context
CIAMO: Context-Intervention-Actor-Mechanism-Outcome
CMO: Context-Mechanism-Outcome
FGD: Focus Group Discussion
FP: Family Planning
HSA: Health Surveillance Assistant
I: Intervention
M: Mechanism
MCM: Modern Contraceptive Method
O: Outcome
PM: Permanent Method
SDG: Sustainable Development Goal
SSI: Semi-Structured Interview
TTV: Tetanus Toxoid Vaccine

## Footnotes

1 Adopted in 2015, the SDGs outline a global call to action to end poverty and to improve the lives of people whilst protecting the planet by 2030. The third SDG focuses on promoting the health and well-being of people everywhere and target 3.7 of this goal relates to sexual and reproductive health-care services, including family planning.
